# Long-term tea consumption reduces the risk of frailty in older Chinese people: Result from a 6-year longitudinal study

**DOI:** 10.3389/fnut.2022.916791

**Published:** 2022-08-15

**Authors:** Tianjing Gao, Siyue Han, Guangju Mo, Qing Sun, Min Zhang, Huaqing Liu

**Affiliations:** ^1^School of Public Health, Bengbu Medical College, Bengbu, China; ^2^School of Health Management, Bengbu Medical College, Bengbu, China

**Keywords:** tea consumption, frailty, China, older people, CLHLS

## Abstract

**Background:**

Vast accumulative evidence suggests that the consumption of tea and its components have various potential health benefits. This study used a longitudinal study to examine the causality between tea consumption and frailty in older Chinese people.

**Methods:**

This study employed the longitudinal data from 2008 to 2014 of the Chinese Longitudinal Healthy Longevity Survey (CLHLS), which were systematically collected through face-to-face interviews. Two thousand six hundred and thirty participants completed six-follow-up surveys in 2014 and were analyzed in this study. The frailty index recommended by Searle and co-authors, including 44 health deficits, was used. A Generalized Estimating Equation (GEE) was applied to determine the risk ratio (RR) with a 95% confidence interval (CI) for frailty, and further subgroup analyses were conducted to investigate whether the risk differed stratified by age, sex, and socioeconomic status. Additionally, the interaction between tea consumption with sex and frailty was tested.

**Results:**

Of the 2,630 participants, 15.3% were consistent daily tea drinkers, and 22.6% reported frailty at the 6-year follow-up. Compared to non-tea drinkers, consistent daily tea drinkers reported a significantly lower ratio of having frailty [risk ratio (RR) = 0.51, 95% confidence interval (CI): 0.36–0.71], adjusting for sociodemographic characteristics, health behavior, socioeconomic status, and chronic illnesses. In further subgroup analyses, consistent daily tea consumption significantly reduced the risk of frailty for males (RR = 0.51, 95% CI: 0.32–0.81) but not females (RR = 0.61, 95% CI: 0.36–1.04); informal education (RR = 0.39, 95% CI: 0.23–0.67) but not formal education (RR = 0.63, 95% CI: 0.39–1.02); financial dependence (RR = 0.40, 95% CI: 0.24–0.65) but not financial independence (RR = 0.66, 95% CI: 0.39–1.12). Tea consumption was associated with a lower risk of frailty in both the young (RR = 0.36, 95% CI: 0.20–0.64) and the oldest (aged ≥ 80) (RR = 0.63, 95% CI: 0.40–0.98). Additionally, females showed a lower tea-mediated risk of frailty in occasional tea consumers (RR = 0.51, 95% CI: 0.29–0.89) and inconsistent tea drinkers (RR = 0.58, 95% CI: 0.37–0.93).

**Conclusions:**

Habitual tea consumption can reduce the risk of frailty in older Chinese, and the benefit varied by age, sex, education, and financial support.

## Introduction

Frailty has become one of the most severe challenges in global public health. A previous study has found that the prevalence of frailty and prefrailty in older adults was 24 and 49%, respectively (1). The rapid expansion of the aging population has caused an increase in the amount of frail older people, which, in turn, has put more pressure on healthcare systems worldwide (2). The prevalence of frailty is higher in older adults but is not considered a part of normal aging (3). Frailty is a complex geriatric syndrome characterized by low resistance and response to stressors, resulting from a general decline in multiple systems and organs (4). Older people who are frail are at risk of increased exposure to adverse outcomes, such as falls, disability, delirium, morbidity, and death (5). Frailty is considered an early stage of disability. Additionally, it has reversible nature, suggesting that appropriate interventions at the right time can prevent, delay, or even reverse this phenomenon (6).

The causes of frailty can be multifaceted and include environmental, physical, and nutrient factors (7). Well-known determinants of frailty are older age, sex (female), lower education, and living without a partner (8). An unhealthy lifestyle is also an essential determinant of frailty, such as smoking, excessive alcohol consumption, poor dietary habits, and low physical activity (9). Previous studies have shown that co-morbidities increase the risk of frailty in older patients (10). Tea is one of the favorite beverages consumed worldwide (11). Vast accumulative evidence suggests that the consumption of tea and its components have various potential health benefits (12), including the prevention of cardiovascular diseases and cancer and its antibacterial, antiangiogenic, antiarthritic, anti-inflammatory, antioxidative, cholesterol-lowering, neuroprotective, and antiviral effects (13). Previous studies have indicated that tea consumption is correlated with a lower risk of stroke, depressive symptoms, and cognitive impairment (14, 15). Additionally, the benefits of tea increase progressively with the frequency of consumption (16). Although many studies have presented the health benefits of tea consumption, others have found the opposite. Different compounds derived from tea, such as caffeine, increase the risk of hypertension (17). Tea drinkers tend to smoke and drink alcohol, while the protective effect of tea consumption is only present in non-smokers or non-alcohol drinkers (18). A previous study has suggested that the positive association between tea consumption and the risk of type 2 diabetes might be caused by pesticide residue in tea leaves (19). Although pesticide residues and mycotoxins could be detected in some tea samples, they are usually below the maximum residue levels. Therefore, tea consumption can be recommended to the public for chronic disease prevention and treatment (20). Although a cross-sectional study has investigated the relationship between green tea consumption and comprehensive frailty in Japanese (21); however, the causality remains unclear.

Drinking tea is a lifestyle and even a culture among Chinese people (22). In China, tea is usually classified as different types according to different manufacturing processes and properties (23), e.g., green tea, black tea and white tea. Green tea is often processed by heating, which inactivates the polyphenol oxidase, oxidative enzymes, and peroxidase ubiquitous in tea leaves (24), and the processes in black tea generally includes four steps, i.e., withering, rolling, fermentation and drying, among which drying plays an important role in fragrance formation and quality fixation of famous high-quality tea (25). Generally, white tea is only processed by prolonged withering and drying processes without any process in enzyme deactivation or fermentation (26). The method of processing and degree of fermentation also affect active ingredients and pro-health properties in tea (27).

This study investigated the causal relationship between tea consumption and frailty among Chinese older adults and the heterogeneity in the association by age groups, sex, and socioeconomic status by using longitudinal data from 2008 to 2014 from the Chinese Longitudinal Healthy Longevity Survey (CLHLS).

## Materials and methods

### Study sample

The data were sourced from the CLHLS, a nationally representative survey jointly conducted by the Center for Healthy Aging and Development Studies at Peking University and Duke University. It was designed to identify the range of environmental, behavioral, social, and biological risk factors impacting healthy longevity; hence, this survey provides detailed information on the family constitution, marriage status, activities of daily living, social activities, health status, and lifestyle for older adults aged ≥ 65 years. More than half of China's counties and cities were selected for the survey (23 out of 31 provinces). The surveyed provinces were Tianjin, Liaoning, Heilongjiang, Jiangsu, Anhui, Jiangxi, Shandong, Hubei, Guangdong, Chongqing, Shanxi, Beijing, Hebei, Shanxi, Jilin, Shanghai, Zhejiang, Fujian, Henan, Hunan, Guangxi, Sichuan, and Hainan (28). The survey area represented 85.3% of the overall population of the country and may be a nationally designated sample (29). The CLHLS systematically collected data from older adults through face-to-face interviews by trained staff (30). The data quality was validated as acceptable, and a more detailed method has been depicted elsewhere (28). The CLHLS study was approved by the Ethics Committee of Peking University (IRB00001052–13074). All participants or their representatives legally signed written consent.

**Figure 1:**
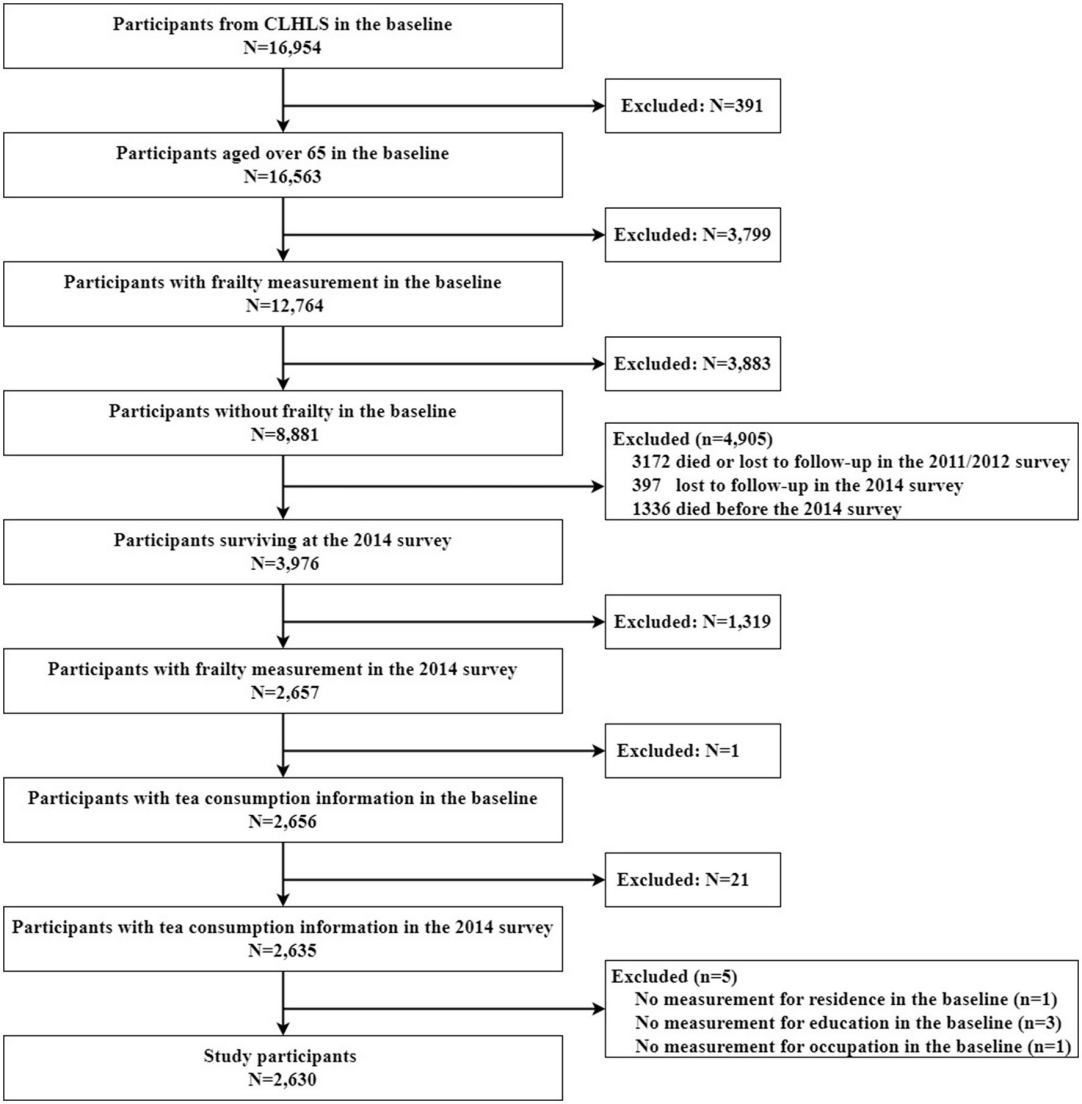
Flow chart of sample selection.

Our study used the 2008–2014 datasets of the CLHLS, which were surveyed from 1 January 2008 to 10 July 2009 and from 10 April 2014 to 23 November 2014, respectively. Of 16,954 respondents in the 2008 baseline interview, 8,881 respondents aged ≥ 65 years had completed data about frailty and had no frailty, with 8,073 being excluded (391 aged <65 years, 3799 had missing data about frailty, and 3,883 had frailty). A total of 4,905 participants were excluded between the two assessments in 2008 and 2014. Of these, 3,172 died or were lost in the 2011 survey, 397 were lost in the 2014 survey, and 1,336 died before the 2014 survey. Finally, 2,630 participants completed a six-follow-up survey in 2014 and were analyzed in this study. The screening procedure is described in Figure 1.

### Definition of frailty

Following the criteria procedure recommended by Searle and co-authors (31), our study used 44 health deficits to form the frailty index (FI), including daily living activities (basic and instrumental), psychological functions, and chronic diseases. The scale of a frailty index in this study has a Cronbach's alpha of 0.870. Each deficit variable was dichotomized or multichotomized and then mapped to a 0–1 interval (e.g., daily task-bathing, where “without assistance” was conferred as 0, “partial assistance” as 0.5, and “need assistance” as 1) to reflect its seriousness. For each participant, the FI scores were measured as the sum of deficit scores divided by the amount of deficit included, ranging from 0 to 1. The participants with a score of >0.21 were referred to as frail (32), while those with a score of ≤0.21 were referred to as not frail. Detailed deficits of the frailty index are shown in Supplementary Table 1.

### Definition of tea consumption

Tea consumption was defined by the answer to the question, “how often do you drink tea at present?” The responses included daily, occasionally, rarely, or never (33). We classified tea consumption into four types based on the information in both 2008 baseline and 2014 follow-up surveys to further capture the uniformity of tea consumption in subsequent years. Participants were defined as “non-tea drinkers” if they reported rarely or never drinking tea both in the baseline and 2014 follow-up surveys, which was the reference classification in the regression analyses; “consistent daily tea drinkers” if they daily drank tea both in the baseline and 2014 follow-up surveys; “consistent tea drinkers” if they daily or occasionally drank tea both in the baseline and 2014 follow-up surveys, but not daily for both; or “inconsistent tea drinkers” if they rarely or never drank tea either in the baseline survey or 2014 follow-up survey but not in both (30).

### Covariates measurement

Some potential covariates were analyzed, including sociodemographic characteristics, socioeconomic status, and health behavior. Sociodemographic characteristics included age group (ages 65–79 years *vs*. ages 80+ years), sex (male *vs*. female), marital status (married *vs*. others), and residence (urban *vs*. rural). Socioeconomic status included education, occupation, and financial support. Education was classified into formal education and informal education, according to ≥1 and <1 year of education, respectively. Occupation before 60 years old was dichotomized into “agricultural work” (coded as 0) and “non-agricultural work” (coded as 1). Financial support has defined as the answer to the question “what is your main source of financial support?,” including financial dependence (coded as 0) and financial independence (coded as 1). Health behavior was measured by current drinking (yes *vs*. no), current smoking (yes *vs*. no), and current exercise (yes *vs*. no). Chronic illnesses were classified as yes and no and included hypertension, diabetes, heart disease, stroke or cardiovascular disease, bronchitis, emphysema, pneumonia, asthma, tuberculosis, cataract, cancer, glaucoma, gastric or duodenal ulcer, Parkinson's disease, bedsore, arthritis, and dementia.

### Statistical analysis

The number and percentage were used to describe the characteristics of tea consumption and frailty, and the difference was verified by the Chi-square test. Mean and standard deviation (SD) were used to describe age and frailty index. The frailty index recommended by Searle and co-authors, including 44 health deficits, was used. A histogram was used to describe the frequency change of the frailty index. A Generalized Estimating Equation (GEE) was applied to determine the risk ratio (RR) with a 95% confidence interval (CI) for frailty, and further subgroup analyses were conducted to investigate whether the risk differed stratified by age, sex, and socioeconomic status. Additionally, the interaction between tea consumption with sex and frailty was tested. All statistical analyses were performed with SPSS 26.0 (Chicago, IL, USA). Statistical significance was considered when *P* was < 0.05 (two-sided).

## Results

Characteristics of the participants at baseline are summarized in Table 1. The sample was composed of 2,630 participants, with 1,353 males (51.4%) and 1,277 females (48.6%). The participants' mean age was 76.84 (SD = 8.5) years. Of these participants, 63.5% were aged 65–79 years, and 36.5% were aged ≥ 80 years; 56.8% were married, 89.1% resided in rural areas, 49.8% had informal education, 68.1% did agricultural work, 57.9% were financially dependent on others, and 51.7% had chronic illnesses.

**Table 1:** Characteristics of older adults by tea consumption, tea consumption status in 2008 baseline, and frailty in the 2014 follow-up.

**Characteristics**	***n* (%)**	**Tea consumption**				**χ^2^**	**Tea consumption status at baseline**			**χ^2^**	**Frailty**	**χ^2^**
		**Consistent daily tea drinkers**	**Consistent tea drinkers**	**Inconsistent tea drinkers**	**Non-tea drinkers**		**Daily**	**Occasionally**	**Rarely or never**			
Age group (years)						3.219				0.630		183.863^***^
65–79	1,671 (63.5)	258 (15.4)	224 (13.4)	605 (36.2)	584 (34.9)		650 (38.9)	273 (16.3)	748 (44.8)		238 (14.2)	
80+	959 (36.5)	144 (15.0)	107 (11.2)	355 (37.0)	353 (36.8)		368 (38.4)	148 (15.4)	443 (46.2)		357 (37.2)	
Sex						167.154^***^				91.247^***^		28.348^***^
Female	1,277 (48.6)	103 (8.1)	119 (9.3)	481 (37.7)	574 (44.9)		379 (29.7)	213 (16.7)	685 (53.6)		346 (27.1)	
Male	1,353 (51.4)	299 (22.1)	212 (15.7)	479 (35.4)	363 (26.8)		639 (47.2)	208 (15.4)	506 (37.4)		249 (18.4)	
Marital status						57.635^***^				27.961^***^		50.101^***^
Married	1,495 (56.8)	281 (18.8)	220 (14.7)	518 (34.6)	476 (31.8)		637 (42.6)	245 (16.4)	613 (41.0)		263 (17.6)	
Others	1,135 (43.2)	121 (10.7)	111 (9.8)	442 (38.9)	461 (40.6)		381 (33.6)	176 (15.5)	578 (50.9)		332 (29.3)	
Residence						11.628^**^				4.458		1.543
Rural	2,344 (89.1)	344 (14.7)	292 (12.5)	850 (36.3)	858 (36.6)		892 (38.1)	375 (16.0)	1,077 (45.9)		522 (22.3)	
Urban	286 (10.9)	58 (20.3)	39 (13.6)	110 (38.5)	79 (27.6)		126 (44.1)	46 (16.1)	114 (39.9)		73 (25.5)	
Education						53.757^***^				20.429^***^		40.655^***^
Formal education	1,319 (50.2)	254 (19.3)	196 (14.9)	452 (34.3)	417 (31.6)		564 (42.8)	210 (15.9)	545 (41.3)		230 (17.4)	
Informal education	1,311 (49.8)	148 (11.3)	135 (10.3)	508 (38.7)	520 (39.7)		454 (34.6)	211 (16.1)	646 (49.3)		365 (27.8)	
Occupation						50.936^***^				39.163^***^		1.430
Agricultural work	1,790 (68.1)	226 (12.6)	200 (11.2)	672 (37.5)	692 (38.7)		622 (34.7)	293 (16.4)	875 (48.9)		393 (22.0)	
Non-agricultural work	840 (31.9)	176 (21.0)	131 (15.6)	288 (34.3)	245 (29.2)		396 (47.1)	128 (15.2)	316 (37.6)		202 (24.0)	
Financial support						51.951^***^				27.456^***^		33.642^***^
Financial dependence	1,523 (57.9)	173 (11.4)	177 (11.6)	584 (38.3)	589 (38.7)		525 (34.5)	258 (16.9)	740 (48.6)		406 (26.7)	
Financial independence	1,107 (42.1)	229 (20.7)	154 (13.9)	376 (34.0)	348 (31.4)		493 (44.5)	163 (14.7)	451 (40.7)		189 (17.1)	
Smoking						75.999^***^				43.123^***^		14.482^***^
Yes	646 (24.6)	148 (22.9)	113 (17.5)	222 (34.4)	163 (25.2)		315 (48.8)	106 (16.4)	225 (34.8)		111 (17.2)	
No	1,984 (75.4)	254 (12.8)	218 (11.0)	738 (37.2)	774 (39.0)		703 (35.4)	315 (15.9)	966 (48.7)		484 (24.4)	
Drinking						67.410^***^				46.404^***^		13.991^***^
Yes	590 (22.4)	144 (24.4)	91 (15.4)	202 (34.2)	153 (25.9)		298 (50.5)	86 (14.6)	206 (34.9)		100 (16.9)	
No	2,040 (77.6)	258 (12.6)	240 (11.8)	758 (37.2)	784 (38.4)		720 (35.3)	335 (16.4)	985 (48.3)		495 (24.3)	
Exercise						17.238^**^				7.937^*^		0.331
Yes	1,030 (39.2)	191 (18.5)	134 (13.0)	372 (36.1)	333 (32.3)		433 (42.0)	157 (15.2)	440 (42.7)		227 (22.0)	
No	1,600 (60.8)	211 (13.2)	197 (12.3)	588 (36.8)	604 (37.8)		585 (36.6)	264 (16.5)	751 (46.9)		368 (23.0)	
Chronic illnesses						3.310				2.732		6.385^*^
Yes	1,361 (51.7)	212 (15.6)	156 (11.5)	500 (36.7)	493 (36.2)		515 (37.8)	209 (15.4)	637 (46.8)		335 (24.6)	
No	1,269 (48.3)	190 (15.0)	175 (13.8)	460 (36.2)	444 (35.0)		503 (39.6)	212 (16.7)	554 (43.7)		260 (20.5)	

* P < 0.05,

** P < 0.01,

*** P < 0.001.

Table 1 summarizes the characteristics of respondents by the types of tea drinkers and tea consumption status at baseline of CLHLS. Of the 2,630 participants, 35.6% were non-tea drinkers, 36.5% were inconsistent tea drinkers, 12.6% were consistent tea drinkers, and 15.3% were consistent daily tea drinkers. Categorized by tea drinking status at baseline, 45.3% were non-tea drinkers, 16.0% were occasional tea drinkers, and 38.7% were daily tea drinkers. Compared to non-tea drinkers, a high frequency of tea drinking was more prevalent among those who were younger, male, married, living in the urban area, doing non-agricultural work, financially independent, doing exercise, and had a formal education. Moreover, tea drinkers tended to smoke and drink. There was no significant difference in tea consumption across age groups and chronic illnesses.

**Figure 2:**
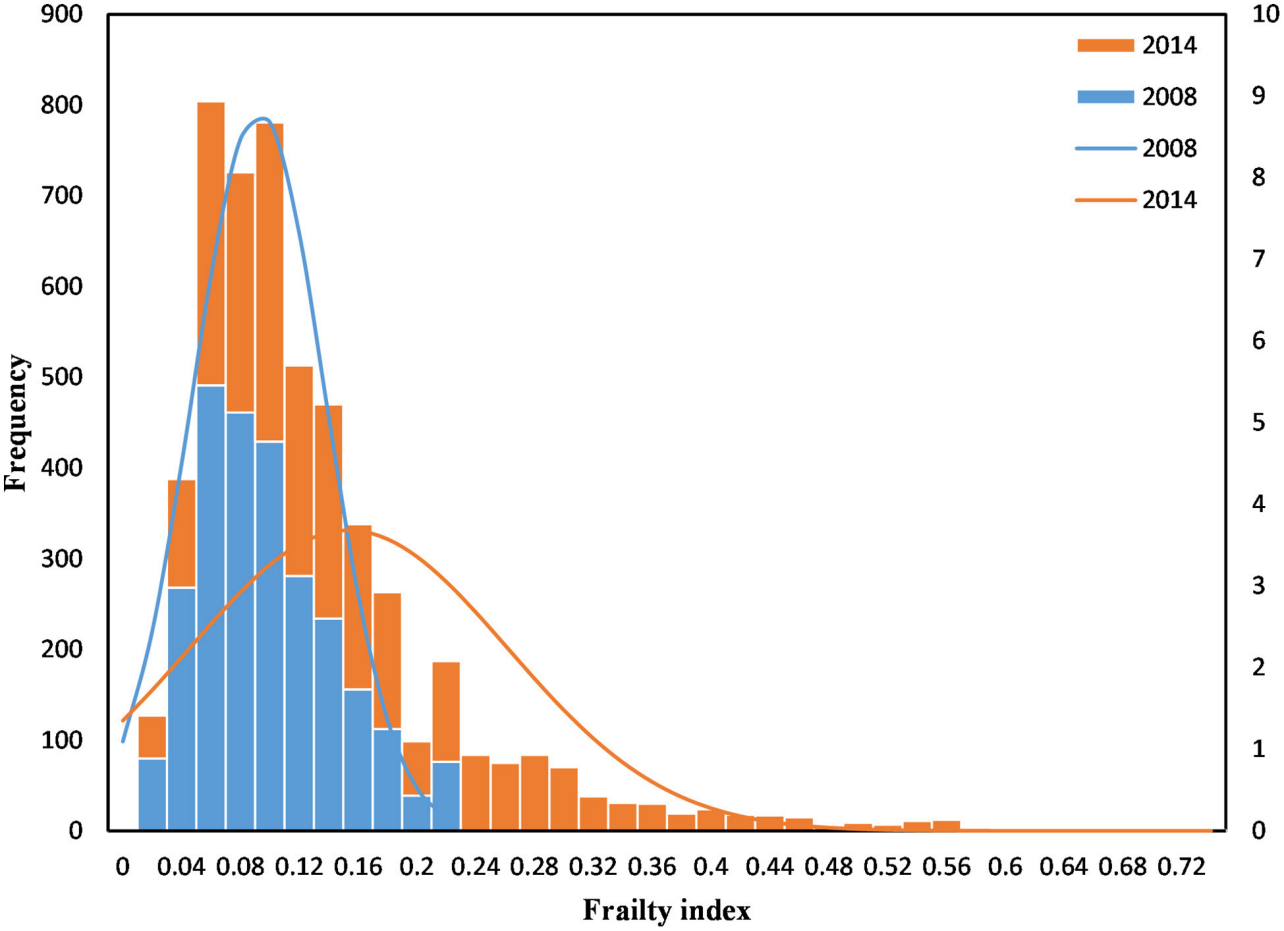
Histogram of the frailty index.

Table 1 also shows the association of participants' characteristics with frailty. The mean frailty index for participants was 0.15 (SD = 0.11) (Figure 2). The prevalence of frailty was 22.6% among older people, higher in the ≥ 80 years group (37.2%) than in the 65–79 years group (14.2%). Older people with frailty were likely to be female, have another marital status, have informal education, not smoke, not drink, be financially dependent on others, and have chronic illnesses. There was no significant difference in residence (*P* = 0.214), occupation (*P* = 0.232), and exercise (*P* = 0.565).

Compared to non-tea drinkers, consistent daily tea drinkers had a significantly lower ratio of frailty (RR = 0.34, 95% CI: 0.29–0.39) in the crude and (RR = 0.51, 95% CI: 0.36–0.71) adjusted models (Table 2). However, daily tea drinkers had a lower ratio of frailty (RR = 0.78, 95% CI: 0.64–0.96) in the crude model, but the difference became small and not statistically significant (RR = 0.86, 95% CI: 0.69–1.07) in the adjusted final model. Additionally, we investigated whether tea benefits differ by age, sex, and socioeconomic status. As shown in Table 3, consistent daily tea consumption resulted in a significantly reduced risk of frailty in males (RR = 0.51, 95% CI: 0.32–0.81) but not in females (RR = 0.61, 95% CI: 0.36–1.04); in informal education (RR = 0.39, 95% CI: 0.23–0.67) but not in formal education (RR = 0.63, 95% CI: 0.39–1.02); in financial dependence (RR = 0.40, 95% CI: 0.24–0.65) but not in financial independence (RR = 0.66, 95% CI: 0.39–1.12). Tea consumption was associated with a lower risk of frailty in both the young (RR = 0.36, 95% CI: 0.20–0.64) and the oldest (aged ≥ 80) (RR = 0.63, 95% CI: 0.40–0.98); agricultural work (RR = 0.53, 95% CI: 0.33–0.83) and non-agricultural work (RR = 0.48, 95% CI: 0.28–0.82).

**Table 2:** Associations between tea consumption and frailty among older Chinese people.

**Characteristics**	**Crude model RR (95% CI)**	**Final model RR (95% CI)**
**Tea consumption (ref**.= **non-tea drinkers)**		
Consistent daily tea drinkers	0.34 (0.29, 0.39)^***^	0.51 (0.36, 0.71)^***^
Consistent tea drinkers	0.44 (0.32, 0.62)^***^	0.99 (0.72, 1.37)
Inconsistent tea drinkers	0.85 (0.63, 1.14)	1.01 (0.81, 1.26)
**Tea consumption status at baseline (ref**.= **rarely or never)**		
Daily	0.78 (0.64, 0.96)^*^	0.86 (0.69, 1.07)
Occasionally	1.03 (0.80, 1.33)	1.11 (0.84, 1.46)

RR represents the risk ratio, 95% CI represents 95% confidence intervals, and Ref. represents reference. ^*^P < 0.05, ^***^P < 0.001. The R2 value for the final model of tea consumption was 0.144. Multiple logistic regression analysis was applied to estimate the RR and 95% CI for frailty. The final model is adjusted for age, sex, marital status, residence, education, occupation, financial support, smoking, drinking, exercise, and chronic illnesses.

**Table 3:** The association between tea consumption and frailty stratified by age, sex, and socioeconomic status.

**Characteristics**	**Consistent daily tea drinkers**	**Consistent tea drinkers**	**Inconsistent tea drinkers**
**Stratified by age group**			
65–79	0.36 (0.20, 0.64)^**^	0.97 (0.63, 1.51)	0.91 (0.66, 1.25)
80+	0.63 (0.40, 0.98)^*^	1.06 (0.66, 1.68)	1.07 (0.79, 1.46)
**Stratified by sex**			
Female	0.61 (0.36, 1.04)	0.95 (0.59, 1.51)	0.81 (0.61, 1.08)
Male	0.51 (0.32, 0.81)^**^	1.18 (0.74, 1.88)	1.38 (0.96, 1.99)
**Stratified by education**			
Formal education	0.63 (0.39, 1.02)	1.17 (0.72, 1.89)	1.21 (0.84, 1.74)
Informal education	0.39 (0.23, 0.67)^**^	0.91 (0.59, 1.42)	0.90 (0.68, 1.19)
**Stratified by occupation**			
Agricultural work	0.53 (0.33, 0.83)^**^	0.82 (0.54, 1.25)	0.91 (0.70, 1.18)
Non-agricultural work	0.48 (0.28, 0.82)^**^	1.33 (0.77, 2.27)	1.24 (0.82, 1.89)
**Stratified by financial support**			
Financial dependence	0.40 (0.24, 0.65)^***^	0.94 (0.63, 1.40)	0.86 (0.66, 1.12)
Financial independence	0.66 (0.39, 1.12)	1.20 (0.68, 2.12)	1.46 (0.98, 2.18)

RR represents the risk ratio, 95% CI represents 95% confidence intervals. ^*^P < 0.05, ^**^P < 0.01, ^***^P < 0.001. The final model is adjusted for age, sex, marital status, residence, education, occupation, financial support, smoking, drinking, exercise, and chronic illnesses.

We tested the interaction between tea consumption and sex to further determine whether tea consumption was differently related to frailty between the sexes (Table 4). The results showed that the effects of tea consumption on frailty were partially mediated by sex. Females showed a lower tea-mediated risk of frailty in occasional tea consumers (RR = 0.51, 95% CI: 0.29–0.89) and inconsistent tea drinkers (RR = 0.58, 95% CI: 0.37–0.93).

**Table 4:** Effect of interaction between tea consumption and sex on frailty.

**Characteristics**	**Crude model RR (95% CI)**	**Final model RR (95% CI)**
**Sex (Ref**.= **Male)**		
Female	1.65 (1.37, 1.98)^***^	1.24 (0.97, 1.59)
**Tea consumption**×**Female**		
Consistent daily tea drinkers		1.17 (0.58, 2.37)
Consistent tea drinkers		0.76 (0.40, 1.47)
Inconsistent tea drinkers		0.58 (0.37, 0.93)^*^
**Tea consumption status at baseline** × **Female**		
Daily		0.84 (0.54, 1.31)
Occasionally		0.51 (0.29, 0.89)^*^

RR represents the risk ratio, 95% CI represents 95% confidence intervals, and Ref. represents reference. ^*^P < 0.05, ^***^P < 0.001. The final model for the interaction between tea consumption and sex had an R2 value of 0.148. Multiple logistic regression analysis was applied to estimate the RR and 95% CI for frailty. The final model is adjusted for age, sex, marital status, residence, education, occupation, financial support, smoking, drinking, exercise, and chronic illnesses.

## Discussion

Our study showed that daily tea consumption was associated with a significantly lower risk of frailty over a 6-year follow-up, and the findings provide new evidence for the protective role of tea consumption in frailty among older people, especially among those who kept regularly drink tea.

The antioxidant status of older people is often poor, and there is evidence that frailty probably results from tissue damage due to oxidative stress and inflammatory processes (34). Tea has been an active component linking anti-inflammatory and antioxidative processes (35); moreover, this anti-inflammatory and antioxidant effect is to inhibit signaling in the inflammatory process by scavenging reactive oxygen species (36). Additionally, tea polyphenols can significantly reduce the pro-inflammatory cytokine tumor necrosis factor, interleukin 6, and interleukin 1β expression, thereby reducing inflammation and achieving the goal of anti-inflammatory (37). A diet with high antioxidant capacity has been strongly inversely correlated with frailty prevalence (38). Tea consumption has been one of the major contributors to dietary antioxidant capacity (39). This beneficial effect on frailty is related to bioactive compounds (21); for example, tea is rich in flavonoids, e.g., epicatechin, catechin, and epigallocatechin gallate. Lakshmi et al. (40) identified that epigallocatechin gallate inhibits activation of nuclear factor-κB, the key inflammatory transcription factor, and performs anti-inflammatory properties. A previous study showed that these bioactive ingredients can relieve inflammation, reduce oxidative stress, and enhance endothelial and cardiomyocyte function (41). Drinking tea is complex as different types of tea have different constitutions and contaminations (42). A previous study has shown that habitual tea consumption is associated with a slightly higher risk of hypertension (43). Although tea has therapeutic properties, it can be a major source of fluoride exposure, and excessive intake of fluoride can lead to health problems, such as fluorosis and skeletal fluorosis (44). Anti-nutritional factors, such as tannins, are often considered responsible for the high incidence of iron deficiency, especially among vulnerable groups (45). A previous study has indicated that tea needs to be consumed frequently enough to reach some cumulative amount to contribute to the health of older adults; however, excessive consumption of tea is not healthy for older adults (46).

Tea consumption is traditionally considered to be a promising non-pharmacological strategy for supplementing the management of hypertension, obesity or diabetes, especially where tea drinking is a widely accepted cultural practice (47). Long-term tea consumption may be necessary to confer health benefits (48). Tea and its components are also extensively used as cardiomyocyte functional foods or dietary supplements for the preventing and treatment of various chronic diseases (49, 50), including preventing cardiovascular disease, Parkinson's disease, metabolic syndromes, osteoporosis, diabetes, obesity, stroke, dementia, and certain cancers (47, 51). Previous studies have shown a negative association between tea consumption and the risk of depression (15). Tea and its bioactive compounds help older people maintain better mental health (52). Tea consumption has been associated with better physical functional performances in older adults (10). Hence, tea consumption may play an essential role in preventing frailty. Habitual tea consumption has been correlated with better health-related quality of life among older Chinese people (53). These results support our data findings of an association between tea consumption and frailty. Tea consumption habits may change over time (54). Perhaps only long-term but not short-term tea drinking behavior plays a beneficial role in frailty.

The prevalence of frailty increases with age (55). The inverse relationship between consistent daily tea consumption and frailty has been statistically significantly different in the young-old people aged 65–79 but not the oldest-old people aged ≥ 80. Tea consumption varied to some extent by age and sex (56). Males and younger older adults tend to drink more tea each day or strong tea with a high concentration of beneficial ingredients; thus, the benefits of tea for these groups may increase (30). The sex gap in frailty increases with advancing age (57). Women have a higher prevalence of frailty than men (58), and it reflected the fact that women suffer a more significant loss of physical reserves and are more likely to experience worse socioeconomic and health conditions than men, indicating a higher chance of fragility (59). Consistent daily tea consumption significantly lessened the risk of having frailty in males but not in females. Previous studies have suggested that women were less likely to be habitual tea drinkers than men (47); the proportion of male and female tea consumers was 72.7 and 55.4%, respectively. Additionally, the balance of daily tea consumers, the duration, and the amount of tea consumption in men were significantly higher than in women (60). Inconsistent tea drinkers and female sex resulted in a reduced risk of frailty. A previous study has shown that women who drank 1–2 cups of tea daily and men who drank ≥ 3 cups of tea daily had a lower prevalence of frailty. Females become frail at a time when they are younger than males. Habitually drinking tea has a powerful antioxidant effect and can prevent frailty in women early (21).

The prevalence of frailty varies among socioeconomic groups (61). Lower economic status, lower levels of education, and multimorbidity have been identified as risk factors for frailty (62). Occupation reflected education, salary, and social status; thus, it has been strongly associated with frailty (63). People with lower income had significantly higher chances of frailty (64). Indeed, the prevalence of smoking and poor diet has already been reported as significantly higher in lower socioeconomic groups (65). Socioeconomic conditions can also influence dietary choices and eating patterns and, therefore, nutritional status (66). Drinking tea may be one of the characteristics of a healthy dietary pattern. Tea consumers have a more nutritious diet with higher protein levels, minerals, and vitamins and fewer added sugars (53). Additionally, tea consumption may also be positively associated with health behavior. For instance, people with higher tea consumption tend to be more health-conscious (67). Health behavior, including regular physical work and exercise, has been significantly associated with a lower risk of frailty (68). Consistent daily tea consumption significantly lessened frailty in those with informal education, agricultural work, and financial dependence. From an economic point of view, a household's budget may not impact the quantity of daily tea consumption (69). Indeed, higher levels of cultural achievement promote an overall healthier lifestyle through good practice derived from regular physical activity, sustained cognitive stimulation, and better social integration (70).

## Strengths and limitations

Tea-drinking behavior may be altered in later years, and the advantages of tea drinking may be long-term. This measurement based on two-time points in the longitudinal study may help capture the role of tea consumption on frailty. We used data from long-term, reliable longitudinal studies to establish that tea consumption was inversely associated with frailty. The study used a simple self-reported screening tool to assess frailty in the older population.

There are several limitations to our study. First, the data for the study came primarily from interviews, which might have had recall bias. Although tea consumption is usually a long-term personal habit, recall bias may be comparatively low. Second, we did not assess the effect of the specific type of tea and its interaction with prescription medication because the relative information was not available in the baseline survey. Finally, there may be other uncontrolled confounders that affected the results.

## Conclusions

Habitual tea consumption can reduce the risk of frailty in older Chinese, and long-term persistence of the habit provides robust protection. In addition, there are age, sex, education, and financial support specific differences in the protective role. Our findings give a further insight into the beneficial role of tea consumption, and have great public health implications for preventing frailty. Future well-designed observational studies on the type of tea and tea intake are needed to fully characterize this association. Additionally, the potential mechanisms, active ingredients in tea, and drug history that may be responsible for the association await further elucidation.

## Recommendations

Tea drinking should be recommended as one of ongoing healthy lifestyle, and is an affordable, cost-effective, and easily adoptable prevention intervention for frailty in older people, especially in males and low socioeconomic status.

## Data availability statement

The original contributions presented in the study are included in the article/Supplementary material, further inquiries can be directed to the corresponding author/s.

## Ethics statement

The CLHLS study was approved by the Research Ethics Committee of Peking University (IRB00001052–13074), and all participants provided written informed consent. All methods were performed in accordance with the relevant guidelines and regulations (e.g., the Declaration of Helsinki).

## Author contributions

MZ and TG: conceptualization. HL, TG, and SH: methodology. HL, QS, and MZ: investigation and data management. TG: original draft preparation. HL, SH, TG, and GM: review and editing. MZ, QS, and SH: supervision. HL, MZ, and GM: project administration. HL and MZ: funding acquisition. All authors contributed to the article and approved the submitted version.

## Funding

This work was supported by the Natural Science Research Project of Anhui Educational Committee (KJ2019A0302) and 512 Talent Training Project of Bengbu Medical College (BY51201203).

## Conflict of interest

The authors declare that the research was conducted in the absence of any commercial or financial relationships that could be construed as a potential conflict of interest.

## Publisher's note

All claims expressed in this article are solely those of the authors and do not necessarily represent those of their affiliated organizations, or those of the publisher, the editors and the reviewers. Any product that may be evaluated in this article, or claim that may be made by its manufacturer, is not guaranteed or endorsed by the publisher.

## Abbreviations

CLHLS: Chinese Longitudinal Healthy Longevity Survey
FI: Frailty Index
RR: Risk Ratio
CI: Confidence Intervals
SD: Standard Deviation
GEE: Generalized Estimating Equation.
