# Corrigendum: Long-term tea consumption reduces the risk of frailty in older Chinese people: Result from a 6-year longitudinal study

**DOI:** 10.3389/fnut.2023.1153726

**Published:** 2023-03-07

**Authors:** Tianjing Gao, Siyue Han, Guangju Mo, Qing Sun, Min Zhang, Huaqing Liu

**Affiliations:** ^1^School of Public Health, Bengbu Medical College, Bengbu, China; ^2^School of Health Management, Bengbu Medical College, Bengbu, China

**Keywords:** tea consumption, frailty, China, older people, CLHLS

In the published article, there was an error in Figures 1, 2, Tables 1–4 and text as published. We erroneously regarded the variable of tea drinking before age 60 as the variable of a current tea drinking to select the sample and analysis the data. We also that we excluded covariate according to the 2008 baseline survey, but 2008 was erroneously written as 2014 in Figure 1. The present final sample size was 2,473, and the corrected sample size was 2,630. The corrected Figures 1, 2, Tables 1–4 and corrected text appear below.

**Figure 1:**
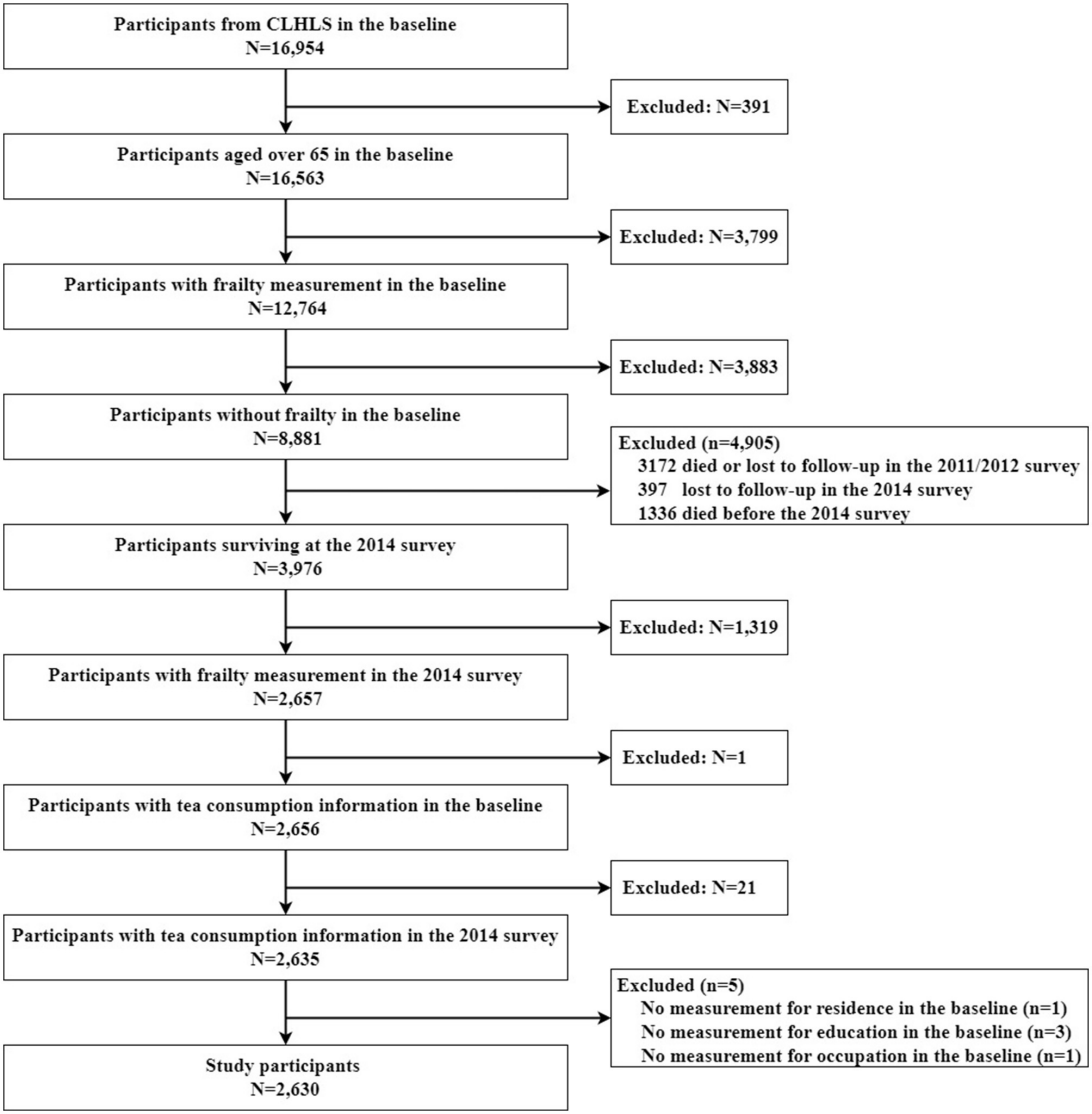
Flow chart of sample selection.

**Figure 2:**
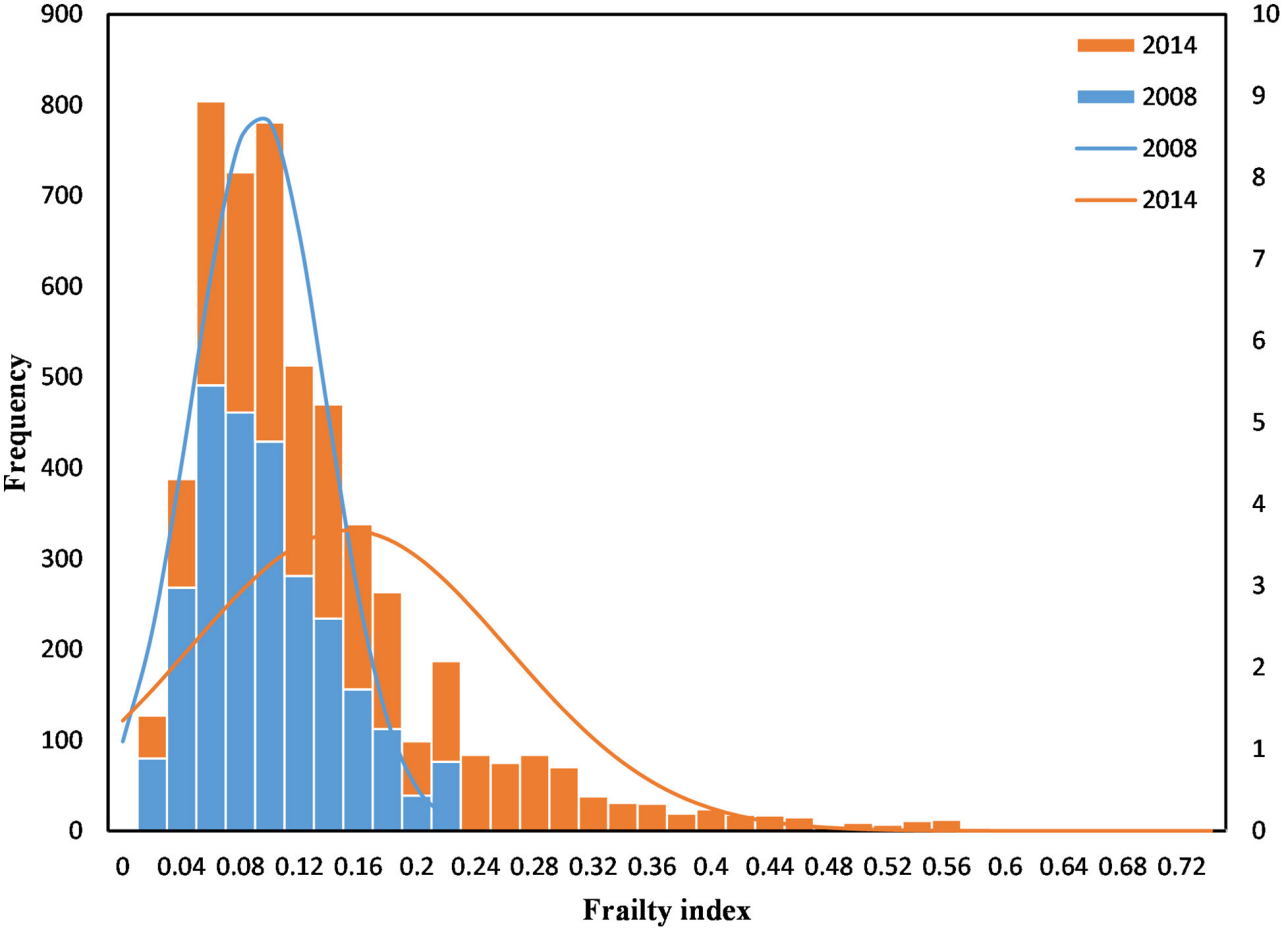
Histogram of the frailty index.

**Table 1:** Characteristics of older adults by tea consumption, tea consumption status in 2008 baseline, and frailty in the 2014 follow-up.

**Characteristics**	***n* (%)**	**Tea consumption**				**χ^2^**	**Tea consumption status at baseline**			**χ^2^**	**Frailty**	**χ^2^**
		**Consistent daily tea drinkers**	**Consistent tea drinkers**	**Inconsistent tea drinkers**	**Non-tea drinkers**		**Daily**	**Occasionally**	**Rarely or never**			
Age group (years)						3.219				0.630		183.863^***^
65–79	1,671 (63.5)	258 (15.4)	224 (13.4)	605 (36.2)	584 (34.9)		650 (38.9)	273 (16.3)	748 (44.8)		238 (14.2)	
80+	959 (36.5)	144 (15.0)	107 (11.2)	355 (37.0)	353 (36.8)		368 (38.4)	148 (15.4)	443 (46.2)		357 (37.2)	
Sex						167.154^***^				91.247^***^		28.348^***^
Female	1,277 (48.6)	103 (8.1)	119 (9.3)	481 (37.7)	574 (44.9)		379 (29.7)	213 (16.7)	685 (53.6)		346 (27.1)	
Male	1,353 (51.4)	299 (22.1)	212 (15.7)	479 (35.4)	363 (26.8)		639 (47.2)	208 (15.4)	506 (37.4)		249 (18.4)	
Marital status						57.635^***^				27.961^***^		50.101^***^
Married	1,495 (56.8)	281 (18.8)	220 (14.7)	518 (34.6)	476 (31.8)		637 (42.6)	245 (16.4)	613 (41.0)		263 (17.6)	
Others	1,135 (43.2)	121 (10.7)	111 (9.8)	442 (38.9)	461 (40.6)		381 (33.6)	176 (15.5)	578 (50.9)		332 (29.3)	
Residence						11.628^**^				4.458		1.543
Rural	2,344 (89.1)	344 (14.7)	292 (12.5)	850 (36.3)	858 (36.6)		892 (38.1)	375 (16.0)	1,077 (45.9)		522 (22.3)	
Urban	286 (10.9)	58 (20.3)	39 (13.6)	110 (38.5)	79 (27.6)		126 (44.1)	46 (16.1)	114 (39.9)		73 (25.5)	
Education						53.757^***^				20.429^***^		40.655^***^
Formal education	1,319 (50.2)	254 (19.3)	196 (14.9)	452 (34.3)	417 (31.6)		564 (42.8)	210 (15.9)	545 (41.3)		230 (17.4)	
Informal education	1,311 (49.8)	148 (11.3)	135 (10.3)	508 (38.7)	520 (39.7)		454 (34.6)	211 (16.1)	646 (49.3)		365 (27.8)	
Occupation						50.936^***^				39.163^***^		1.430
Agricultural work	1,790 (68.1)	226 (12.6)	200 (11.2)	672 (37.5)	692 (38.7)		622 (34.7)	293 (16.4)	875 (48.9)		393 (22.0)	
Non-agricultural work	840 (31.9)	176 (21.0)	131 (15.6)	288 (34.3)	245 (29.2)		396 (47.1)	128 (15.2)	316 (37.6)		202 (24.0)	
Financial support						51.951^***^				27.456^***^		33.642^***^
Financial dependence	1,523 (57.9)	173 (11.4)	177 (11.6)	584 (38.3)	589 (38.7)		525 (34.5)	258 (16.9)	740 (48.6)		406 (26.7)	
Financial independence	1,107 (42.1)	229 (20.7)	154 (13.9)	376 (34.0)	348 (31.4)		493 (44.5)	163 (14.7)	451 (40.7)		189 (17.1)	
Smoking						75.999^***^				43.123^***^		14.482^***^
Yes	646 (24.6)	148 (22.9)	113 (17.5)	222 (34.4)	163 (25.2)		315 (48.8)	106 (16.4)	225 (34.8)		111 (17.2)	
No	1,984 (75.4)	254 (12.8)	218 (11.0)	738 (37.2)	774 (39.0)		703 (35.4)	315 (15.9)	966 (48.7)		484 (24.4)	
Drinking						67.410^***^				46.404^***^		13.991^***^
Yes	590 (22.4)	144 (24.4)	91 (15.4)	202 (34.2)	153 (25.9)		298 (50.5)	86 (14.6)	206 (34.9)		100 (16.9)	
No	2,040 (77.6)	258 (12.6)	240 (11.8)	758 (37.2)	784 (38.4)		720 (35.3)	335 (16.4)	985 (48.3)		495 (24.3)	
Exercise						17.238^**^				7.937^*^		0.331
Yes	1,030 (39.2)	191 (18.5)	134 (13.0)	372 (36.1)	333 (32.3)		433 (42.0)	157 (15.2)	440 (42.7)		227 (22.0)	
No	1,600 (60.8)	211 (13.2)	197 (12.3)	588 (36.8)	604 (37.8)		585 (36.6)	264 (16.5)	751 (46.9)		368 (23.0)	
Chronic illnesses						3.310				2.732		6.385^*^
Yes	1,361 (51.7)	212 (15.6)	156 (11.5)	500 (36.7)	493 (36.2)		515 (37.8)	209 (15.4)	637 (46.8)		335 (24.6)	
No	1,269 (48.3)	190 (15.0)	175 (13.8)	460 (36.2)	444 (35.0)		503 (39.6)	212 (16.7)	554 (43.7)		260 (20.5)	

* P < 0.05,

** P < 0.01,

*** P < 0.001.

**Table 2:** Associations between tea consumption and frailty among older Chinese people.

**Characteristics**	**Crude model RR (95% CI)**	**Final model RR (95% CI)**
**Tea consumption (ref**.= **non-tea drinkers)**		
Consistent daily tea drinkers	0.34 (0.29, 0.39)^***^	0.51 (0.36, 0.71)^***^
Consistent tea drinkers	0.44 (0.32, 0.62)^***^	0.99 (0.72, 1.37)
Inconsistent tea drinkers	0.85 (0.63, 1.14)	1.01 (0.81, 1.26)
**Tea consumption status at baseline (ref**.= **rarely or never)**		
Daily	0.78 (0.64, 0.96)^*^	0.86 (0.69, 1.07)
Occasionally	1.03 (0.80, 1.33)	1.11 (0.84, 1.46)

RR represents the risk ratio, 95% CI represents 95% confidence intervals, and Ref. represents reference. ^*^P < 0.05, ^***^P < 0.001. The R2 value for the final model of tea consumption was 0.144. Multiple logistic regression analysis was applied to estimate the RR and 95% CI for frailty. The final model is adjusted for age, sex, marital status, residence, education, occupation, financial support, smoking, drinking, exercise, and chronic illnesses.

**Table 3:** The association between tea consumption and frailty stratified by age, sex, and socioeconomic status.

**Characteristics**	**Consistent daily tea drinkers**	**Consistent tea drinkers**	**Inconsistent tea drinkers**
**Stratified by age group**			
65–79	0.36 (0.20, 0.64)^**^	0.97 (0.63, 1.51)	0.91 (0.66, 1.25)
80+	0.63 (0.40, 0.98)^*^	1.06 (0.66, 1.68)	1.07 (0.79, 1.46)
**Stratified by sex**			
Female	0.61 (0.36, 1.04)	0.95 (0.59, 1.51)	0.81 (0.61, 1.08)
Male	0.51 (0.32, 0.81)^**^	1.18 (0.74, 1.88)	1.38 (0.96, 1.99)
**Stratified by education**			
Formal education	0.63 (0.39, 1.02)	1.17 (0.72, 1.89)	1.21 (0.84, 1.74)
Informal education	0.39 (0.23, 0.67)^**^	0.91 (0.59, 1.42)	0.90 (0.68, 1.19)
**Stratified by occupation**			
Agricultural work	0.53 (0.33, 0.83)^**^	0.82 (0.54, 1.25)	0.91 (0.70, 1.18)
Non-agricultural work	0.48 (0.28, 0.82)^**^	1.33 (0.77, 2.27)	1.24 (0.82, 1.89)
**Stratified by financial support**			
Financial dependence	0.40 (0.24, 0.65)^***^	0.94 (0.63, 1.40)	0.86 (0.66, 1.12)
Financial independence	0.66 (0.39, 1.12)	1.20 (0.68, 2.12)	1.46 (0.98, 2.18)

RR represents the risk ratio, 95% CI represents 95% confidence intervals. ^*^P < 0.05, ^**^P < 0.01, ^***^P < 0.001. The final model is adjusted for age, sex, marital status, residence, education, occupation, financial support, smoking, drinking, exercise, and chronic illnesses.

**Table 4:** Effect of interaction between tea consumption and sex on frailty.

**Characteristics**	**Crude model RR (95% CI)**	**Final model RR (95% CI)**
**Sex (Ref**.= **Male)**		
Female	1.65 (1.37, 1.98)^***^	1.24 (0.97, 1.59)
**Tea consumption**×**Female**		
Consistent daily tea drinkers		1.17 (0.58, 2.37)
Consistent tea drinkers		0.76 (0.40, 1.47)
Inconsistent tea drinkers		0.58 (0.37, 0.93)^*^
**Tea consumption status at baseline** × **Female**		
Daily		0.84 (0.54, 1.31)
Occasionally		0.51 (0.29, 0.89)^*^

RR represents the risk ratio, 95% CI represents 95% confidence intervals, and Ref. represents reference. ^*^P < 0.05, ^***^P < 0.001. The final model for the interaction between tea consumption and sex had an R2 value of 0.148. Multiple logistic regression analysis was applied to estimate the RR and 95% CI for frailty. The final model is adjusted for age, sex, marital status, residence, education, occupation, financial support, smoking, drinking, exercise, and chronic illnesses.

A correction has been made to the Section **Abstract**, subsection “*Methods*.” This sentence previously stated:

“Two thousand four hundred and seventy three participants completed six-follow-up surveys in 2014 and were analyzed in this study.”

The corrected sentence appears below:

“Two thousand six hundred and thirty participants completed six-follow-up surveys in 2014 and were analyzed in this study. ”

A correction has been made to the Section **Abstract**, subsection “*Results*.” These sentences previously stated:

“Of the 2,473 participants, 14.1% were consistent daily tea drinkers, and 22.6% reported frailty at the 6-year follow-up. Compared to non-tea drinkers, consistent daily tea drinkers reported a significantly lower ratio of having frailty [risk ratio (RR) = 0.54, 95% confidence interval (CI): 0.38–0.78], adjusting for sociodemographic characteristics, health behavior, socioeconomic status, and chronic illnesses. In further subgroup analyses, consistent daily tea consumption significantly reduced the risk of frailty for males (RR = 0.53, 95% CI: 0.32–0.87) but not females (RR = 0.65, 95% CI: 0.37–1.12); in the young (RR = 0.40, 95% CI: 0.22–0.74) but not in the oldest (aged ≥ 80) (RR = 0.66, 95% CI: 0.40–1.06); informal education (RR = 0.48, 95% CI: 0.28–0.84) but not formal education (RR = 0.62, 95% CI: 0.37–1.03); financial dependence (RR = 0.42, 95% CI: 0.25–0.71) but not financial independence (RR = 0.71, 95% CI: 0.41–1.23).”

The corrected sentences appear below:

“Of the 2,630 participants, 15.3% were consistent daily tea drinkers, and 22.6% reported frailty at the 6-year follow-up. Compared to non-tea drinkers, consistent daily tea drinkers reported a significantly lower ratio of having frailty [risk ratio (RR) = 0.51, 95% confidence interval (CI): 0.36–0.71], adjusting for sociodemographic characteristics, health behavior, socioeconomic status, and chronic illnesses. In further subgroup analyses, consistent daily tea consumption significantly reduced the risk of frailty for males (RR = 0.51, 95% CI: 0.32–0.81) but not females (RR = 0.61, 95% CI: 0.36–1.04); informal education (RR = 0.39, 95% CI: 0.23–0.67) but not formal education (RR = 0.63, 95% CI: 0.39–1.02); financial dependence (RR = 0.40, 95% CI: 0.24–0.65) but not financial independence (RR = 0.66, 95% CI: 0.39–1.12). Tea consumption was associated with a lower risk of frailty in both the young (RR = 0.36, 95% CI: 0.20–0.64) and the oldest (aged ≥ 80) (RR = 0.63, 95% CI: 0.40–0.98).”

A correction has been made to the Section **Materials and methods**, subsection “*Study sample*,” paragraph 2. This sentence previously stated:

“Finally, 2,473 participants completed a six-follow-up survey in 2014 and were analyzed in this study.”

The corrected sentence appears below:

“Finally, 2,630 participants completed a six-follow-up survey in 2014 and were analyzed in this study.”

A correction has been made to the Section **Materials and methods**, subsection “*Definition of frailty*,” paragraph 1. This sentence previously stated:

“The scale of a frailty index in this study has a Cronbach's alpha of 0.867.”

The corrected sentence appears below:

“The scale of a frailty index in this study has a Cronbach's alpha of 0.870.”

A correction has been made to the Section **Materials and methods**, subsection “*Definition of frailty*,” paragraph 1. This sentence previously stated:

“The participants with a score of >0.21 were referred to as frail (32), while those with a score of <0.21 were referred to as not frail.”

The corrected sentence appears below:

“The participants with a score of >0.21 were referred to as frail (32), while those with a score of ≤0.21 were referred to as not frail.”

Corrections have been made to the Section **Results**, paragraphs 1, 2, 3 and 4. These sentences previously stated:

“Characteristics of the participants at baseline are summarized in Table 1. The sample was composed of 2,473 participants, with 1,251 males (50.6%) and 1,222 females (49.4%). The participants' mean age was 76.88 (SD = 8.5) years. Of these participants, 63.4% were aged 65–79 years, and 36.6% were aged ≥ 80 years; 56.1% were married, 89.1% resided in rural areas, 50.5% had informal education, 68.4% did agricultural work, 58.1% were financially dependent on others, and 51.7% had chronic illnesses.”

“Table 1 summarizes the characteristics of respondents by the types of tea drinkers and tea consumption status at baseline of CLHLS. Of the 2,473 participants, 35.8% were non-tea drinkers, 38.8% were inconsistent tea drinkers, 11.2% were consistent tea drinkers, and 14.1% were consistent daily tea drinkers. Categorized by tea drinking status at baseline, 48.2% were non-tea drinkers, 15.6% were occasional tea drinkers, and 36.2% were daily tea drinkers.”

“Table 1 also shows the association of participants' characteristics with frailty. The mean frailty index for participants was 0.15 (SD = 0.11) (Figure 2). The prevalence of frailty was 22.6% among older people, higher in the ≥ 80 years group (37.0%) than in the 65–79 years group (14.3%). Older people with frailty were likely to be female, have another marital status, have informal education, not smoke, not drink, be financially dependent on others, and have chronic illnesses. There was no significant difference in residence (*P* = 0.156), occupation (*P* = 0.381), and exercise (*P* = 0.392).”

“Compared to non-tea drinkers, consistent daily tea drinkers had a significantly lower ratio of frailty (RR = 0.47, 95% CI: 0.33–0.66) in the crude and (RR = 0.54, 95% CI: 0.38–0.78) adjusted models (Table 2). However, daily tea drinkers had a lower ratio of frailty (RR = 0.75, 95% CI: 0.60–0.92) in the crude model, but the difference became small and not statistically significant (RR = 0.81, 95% CI: 0.65–1.02) in the adjusted final model. Additionally, we investigated whether tea benefits differ by age, sex, and socioeconomic status. As shown in Table 3, consistent daily tea consumption resulted in a significantly reduced risk of frailty in males (RR = 0.53, 95% CI: 0.32–0.87) but not in females (RR = 0.65, 95% CI: 0.37–1.12); in the young (RR = 0.40, 95% CI: 0.22–0.74) but not in the oldest (aged ≥ 80) (RR = 0.66, 95% CI: 0.40–1.06); in informal education (RR = 0.48, 95% CI: 0.28–0.84) but not in formal education (RR = 0.62, 95% CI: 0.37–1.03); in financial dependence (RR = 0.42, 95% CI: 0.25–0.71) but not in financial independence (RR = 0.71, 95% CI: 0.41–1.23). Tea consumption was associated with a lower risk of frailty in both agricultural work (RR = 0.61, 95% CI: 0.38–0.97) and non-agricultural work (RR = 0.46, 95% CI: 0.25–0.82).”

The corrected sentences appear below:

“Characteristics of the participants at baseline are summarized in Table 1. The sample was composed of 2,630 participants, with 1,353 males (51.4%) and 1,277 females (48.6%). The participants' mean age was 76.84 (SD = 8.5) years. Of these participants, 63.5% were aged 65–79 years, and 36.5% were aged ≥ 80 years; 56.8% were married, 89.1% resided in rural areas, 49.8% had informal education, 68.1% did agricultural work, 57.9% were financially dependent on others, and 51.7% had chronic illnesses.”

“Table 1 summarizes the characteristics of respondents by the types of tea drinkers and tea consumption status at baseline of CLHLS. Of the 2,630 participants, 35.6% were non-tea drinkers, 36.5% were inconsistent tea drinkers, 12.6% were consistent tea drinkers, and 15.3% were consistent daily tea drinkers. Categorized by tea drinking status at baseline, 45.3% were non-tea drinkers, 16.0% were occasional tea drinkers, and 38.7% were daily tea drinkers.”

“Table 1 also shows the association of participants' characteristics with frailty. The mean frailty index for participants was 0.15 (SD = 0.11) (Figure 2). The prevalence of frailty was 22.6% among older people, higher in the ≥ 80 years group (37.2%) than in the 65–79 years group (14.2%). Older people with frailty were likely to be female, have another marital status, have informal education, not smoke, not drink, be financially dependent on others, and have chronic illnesses. There was no significant difference in residence (*P* = 0.214), occupation (*P* = 0.232), and exercise (*P* = 0.565).”

“Compared to non-tea drinkers, consistent daily tea drinkers had a significantly lower ratio of frailty (RR = 0.34, 95% CI: 0.29–0.39) in the crude and (RR = 0.51, 95% CI: 0.36–0.71) adjusted models (Table 2). However, daily tea drinkers had a lower ratio of frailty (RR = 0.78, 95% CI: 0.64–0.96) in the crude model, but the difference became small and not statistically significant (RR = 0.86, 95% CI: 0.69–1.07) in the adjusted final model. Additionally, we investigated whether tea benefits differ by age, sex, and socioeconomic status. As shown in Table 3, consistent daily tea consumption resulted in a significantly reduced risk of frailty in males (RR = 0.51, 95% CI: 0.32–0.81) but not in females (RR = 0.61, 95% CI: 0.36–1.04); in informal education (RR = 0.39, 95% CI: 0.23–0.67) but not in formal education (RR = 0.63, 95% CI: 0.39–1.02); in financial dependence (RR = 0.40, 95% CI: 0.24–0.65) but not in financial independence (RR = 0.66, 95% CI: 0.39–1.12). Tea consumption was associated with a lower risk of frailty in both the young (RR = 0.36, 95% CI: 0.20–0.64) and the oldest (aged ≥ 80) (RR = 0.63, 95% CI: 0.40–0.98); agricultural work (RR = 0.53, 95% CI: 0.33–0.83) and non-agricultural work (RR = 0.48, 95% CI: 0.28–0.82).”

The authors apologize for these errors and state that these do not change the scientific conclusions of the article in any way. The original article has been updated.

