# Supplementary material for: Long-term tea consumption reduces the risk of frailty in older Chinese people: Result from a 6-year longitudinal study
Source: Front Nutr. 2022 Aug 15;9:916791. doi: 10.3389/fnut.2022.916791 (PMC9421071; doi:10.3389/fnut.2022.916791)
Supplement: Supplementary file 1 [file Table_1.docx]

Supplementary Material

# Supplementary Table

**Supplementary Table 1.** List of items included in a frailty index

| Number | Variables | Values |
| --- | --- | --- |
| 1 | Feel useless with age | Never =0; seldom =0.25; sometimes =0.5; often =0.75; always=1 |
| 2 | Feel lonely and isolated | Never =0; seldom =0.25; sometimes =0.5; often =0.75; always=1 |
| 3 | Feel fearful or anxious | Never =0; seldom =0.25; sometimes =0.5; often =0.75; always=1 |
| 4 | Keep my belongings neat and clean | Always =0; often =0.25; sometimes =0.5; seldom =0.75; never=1 |
| 5 | Self-reported health | Very good =0; good =0.25; so so=0.5; bad =0.75; very bad =1 |
| 6 | Do you feel any change of your health | Much better =0; a little better =0.25; no change =0.5;a little worse |
|  | since last year? | =0.75; much worse=1 |
| 7 | Make own decision | Always =0; often =0.25; sometimes =0.5; seldom =0.75; never=1 |
| 8 | Bathing | without assistance =0; one part assistance =0.5; more than one part assistance=1 |
| 9 | Dressing | without assistance =0;need assistance for trying shoes =0.5; |
|  |  | assistance in getting clothes and getting dressed=1 |
| 10 | Toileting | without assistance =0;assistance in cleaning or arranging clothes |
|  |  | =0.5; don't use toilet=1 |
| 11 | Transferring | without assistance =0;with assistance =0.5; bedridden=1 |
| 12 | Continence | without assistance =0;occasional accidents =0.5; incontinent=1 |
| 13 | Feeding | without assistance =0;with some help =0.5; need feeding=1 |
| 14 | Able to go outside to visit neighbors? | Yes =0; a little difficult =0.5; unable to do so=1 |
| 15 | Able to go shopping by yourself? | Yes =0; a little difficult =0.5; unable to do so=1 |
| 16 | Able to make food by yourself? | Yes =0; a little difficult =0.5; unable to do so=1 |
| 17 | Able to wash clothes by yourself? | Yes =0; a little difficult =0.5; unable to do so=1 |
| 18 | Able to walk one kilometer? | Yes =0; a little difficult =0.5; unable to do so=1 |
| 19 | Able to carry 5kg weight? | Yes =0; a little difficult =0.5; unable to do so=1 |
| 20 | Able to crouch and stand three times? | Yes =0; a little difficult =0.5; unable to do so=1 |
| 21 | Able to take public transportation? | Yes =0; a little difficult =0.5; unable to do so=1 |
| 22 | Visual function | can see and distinguish =0; can see only =0.33; can't see =0.67; |
|  |  | blind =1 |
| 23 | Hand behind neck | Both hands=0; right hand =0.5; left hand =0.5; neither hand=1 |
| 24 | Hand behind lower back | Both hands=0; right hand =0.5; left hand =0.5; neither hand=1 |
| 25 | Raise arms upright | Two arms=0; right arm =0.5; left arm =0.5; neither left nor right |
|  |  | arms=1 |
| 26 | Able to stand up from sitting in a chair? | yes, without using hands =0; yes, using hands =0.5; no =1 |
| 27 | Able to pick up a book from the floor? | yes, standing =0; yes, sitting =0.5; no=1 |
| 28 | Of times suffering from serious illness | no serious illness =0; one serious illness =l; two or more serious |
|  | within the past two years | illnesses =2 |
| 29 | Suffering from hypertension? | No =0; yes=1 |
| 30 | Suffering from diabetes? | No =0; yes=1 |
| 31 | Suffering from heart disease? | No =0; yes=1 |
| 32 | Suffering from stroke or cardiovascular | No =0; yes=1 |
|  | disease? |  |
| 33 | Suffering from bronchitis, emphysema, | No =0; yes=1 |
|  | pneumonia, asthma? |  |
| 34 | Suffering from tuberculosis? | No =0; yes=1 |
| 35 | Suffering from cataract? | No =0; yes=1 |
| 36 | Suffering from cancer? | No =0; yes=1 |
| 37 | Suffering from glaucoma? | No =0; yes=1 |
| 38 | Suffering from gastric or duodenal ulcer? | No =0; yes=1 |
| 39 | Suffering from parkinson's disease? | No =0; yes=1 |
| 40 | Suffering from bedsore? | No =0; yes=1 |
| 41 | Suffering from arthritis? | No =0; yes=1 |
| 42 | Suffering from dementia? | No =0; yes=1 |
| 43 | Was interviewee able to hear? | yes, without hearing aid =0; yes, but needs hearing aid =0.33; |
|  |  | partly, despite hearing aid =0.67;no=1 |
| 44 | The health of interviewee rated by | surprisingly healthy =0; relatively healthy =0.33; moderately ill |
|  | interviewer | =0.67; very ill=l |
